# Continuous formation of *N*-chloro-*N*,*N*-dialkylamine solutions in well-mixed meso-scale flow reactors

**DOI:** 10.3762/bjoc.11.262

**Published:** 2015-12-02

**Authors:** A John Blacker, Katherine E Jolley

**Affiliations:** 1 Institute of Process Research and Development, School of Chemistry and School of Chemical and Process Engineering, University of Leeds, Woodhouse Lane, Leeds, LS2 9JT, Leeds, UK

**Keywords:** amine, biphasic, chloramine, chlorination, continuous flow chemistry, CSTR, static mixer, sodium hypochlorite, tube reactor

## Abstract

The continuous flow synthesis of a range of organic solutions of *N*,*N*-dialkyl-*N*-chloramines is described using either a bespoke meso-scale tubular reactor with static mixers or a continuous stirred tank reactor. Both reactors promote the efficient mixing of a biphasic solution of *N*,*N*-dialkylamine in organic solvent, and aqueous sodium hypochlorite to achieve near quantitative conversions, in 72–100% in situ yields, and useful productivities of around 0.05 mol/h with residence times from 3 to 20 minutes. Initial calorimetric studies have been carried out to inform on reaction exotherms, rates and safe operation. Amines which partition mainly in the organic phase require longer reaction times, provided by the CSTR, to compensate for low mass transfer rates in the biphasic system. The green metrics of the reaction have been assessed and compared to existing procedures and have shown the continuous process is improved over previous procedures. The organic solutions of *N*,*N*-dialkyl-*N*-chloramines produced continuously will enable their use in tandem flow reactions with a range of nucleophilic substrates.

## Introduction

*N*-Chloramines provide a versatile and reactive class of reagents for use in electrophilic amination and other reactions. *N*-Chloro-*N*,*N*-dialkylamines have been shown to offer a broad range of products from reactions with i) unsaturated C–C bonds to give amines [1–3] and heterocycles [1,4]; ii) Grignard and organozinc reagents to give amines [1]; iii) aldehydes to give amides [5–7]; iv) base to give imines [8]; v) alkyl and aryl C–H bonds in the presence of acid and visible light to form heterocycles [9–10]. Furthermore they have also been used for chlorination of aromatics in the presence of acid [11], and as reagents in the syntheses of natural products [12–13].

Despite their versatility as reagents, the exothermicity and instability that occurs in both their formation and reaction, has reduced the interest in their use for reasons of process safety. Isolation of many *N*-chloramines is unadvisable with reports of unpredictable and rapid decomposition [11,14–16].

*N*-Chloramines are prepared conventionally by reaction of the amine precursor with an electrophilic chlorine source [14]. Despite its atom efficiency use of chlorine gas is undesirable due to its toxicity and strong oxidising properties, making it highly hazardous and operationally difficult to use. In addition, the hydrochloric acid byproduct requires additional processing to neutralise and separate it. *N*-Chlorosuccinimide is used frequently in the laboratory because it is a relatively stable solid that is easily weighed and added to reactions [3,17], however, it has a low atom economy, poor economics, and requires separation of the succinimide byproduct [18]. The *tert*-butyl hypochlorite (*t-*BuOCl) reagent is also used regularly, but is similarly expensive, wasteful and hazardous [14,19–21]. On the other hand sodium hypochlorite (NaOCl) is an inexpensive byproduct of chlorine manufacture, and offers a safer, greener source of electrophilic chlorine for producing *N*-chloramines [11,14,22]. Reaction times can, however, be long [14,23–24], and yields low [25]. Zhong et al. have reported a process using NaOCl to generate *t-*BuOCl in situ for chloramine formation, applying the methodology to a broad range of substrates in high yields [20].

Published literature on chloramine formation is limited to batch procedures, however, the use of continuous processes could offer significant advantages. Use of continuous flow reactors to achieve steady-state allows precise control over the reaction parameters to improve safety, selectivity, productivity and consistency. Reduced reactor volumes compared to conventional batch reactors limit the quantity of reacting material at any one time; particularly important for the preparation of hazardous or unstable products. Furthermore smaller volumes with higher surface areas enable faster heat removal and better temperature control to limit unwanted side reactions. Further kinetic control is achieved with reactant concentrations, feed rates, mixing regime and residence time resulting in higher product yields.

**Scheme 1:**
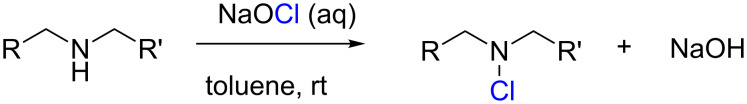
Two-phase reaction of *N*,*N*-dialkylamine and sodium hypochlorite.

Herein we report the use of different types of bespoke continuous reactors for preparing different *N*-chloro-*N*,*N*-dialkylamines. The use of these materials in forming a range of different nitrogen containing products via a cascade/tandem procedure will be reported elsewhere. This atom efficient procedure, with sodium hydroxide and water the only byproducts, should have better green metrics than other methods. Using biphasic reaction conditions with the amine dissolved in a water immiscible, organic solvent such as toluene enabled facile separation of the organic soluble product from the water soluble NaOH (Scheme 1). Such separation of biphasic mixtures is known in continuous systems, for example membrane-based separators [26–27].

Mixing is a key parameter for reactions in multiphasic systems and characterisation of material flows within different continuous reactors is widely studied by both modelling and experiment [28–29]. The rate of reaction of NaOCl and amines at high pH is very fast with a second order rate constant, *k_obs_* of 1.52 × 10^5^ L·mol^−1^·min^−1^ reported for dimethylamine [30]. In this case it is the rate of mass transfer of the reagents, partitioned between the two liquid phases that limits the rate of product formation, rather than the chemical rate of reaction between the two species. Continuous liquid biphasic reactions are usually poorly mixed within micro-scale reactors, as low fluid velocities and frictional wall effects cause laminar flow and phase separation into alternating organic–aqueous segmented flow; whilst meso-scale reactors are much better suited, and provide more accurate and reliable information for scale-up. Interaction of the reactants occurs only at the phase interface, so efficient mixing increases the surface area and promotes product formation. Within batch or continuous stirred tank reactors an increase in mixing intensity is achieved with optimised impellor design and speed, alongside a reactor designed to disrupt the flow and increase turbulence. Within meso-scale tubular reactors the addition of in-line aids such as split and recombination streams [31–32], or static mixers [32–33], can enhance mixing and mass transfer between phases. Phase-transfer catalysts (PTCs) can also be used to promote reactions across phase boundaries [31], their use would, however, incur additional financial costs for the reaction, and also the need for purification and removal of the catalyst from the reaction solution.

## Results and Discussion

### Calorimetry

**Figure 1:**
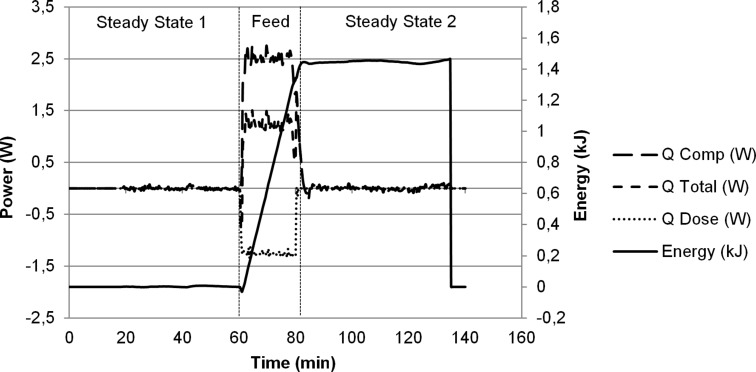
Calorimeter trace for the single phase reaction of morpholine (aq) and NaOCl (aq). *Q* Comp: compensatory power, *Q* Total: total power, *Q* Dose: power delivered by dosing of room temperature NaOCl (aq) to cooled reaction solution. Energy: heat energy.

In view of the potential dangers involved in making chloramines we began our studies with calorimetric analysis. The formation *N*-chloromorpholine was studied by feeding 1.1 M NaOCl (aq) into a 1 M morpholine (aq) at a rate of 1 g/min. The calorimeter jacket temperature was set at −15 °C, and power compensation used to maintain the reactor at 5 °C. 20 mL of the NaOCl solution were added to 20 mmol of morpholine over 20 min, and after subtracting the feed solution temperature (*Q* Dose) from compensatory power (*Q* Comp) the total was 2.76 W. For this calibrated calorimeter this equates to −1.47 kJ which gives a heat of reaction, Δ*H*_r_ = −73.4 kJ·mol^−1^ for the formation of *N*-chloromorpholine (Figure 1). Calculation of the heat of reaction by bond forming/bond breaking calculation, according to mechanisms proposed in the literature, give a Δ*H*_r_ of −72 kJ·mol^−1^ (Supporting Information File 1) [30].

Figure 1 shows the calorimetric trace for the formation of *N*-chloromorpholine. Following the addition of NaOCl (aq) there is a small delay in the reaction which may be mixing related. A rapid exotherm then occurs with 1.37 kJ energy released continuously over the 20 minute feed. When the NaOCl (aq) addition is stopped there is a further release of 0.09 kJ energy over 2 minutes indicating a small accumulation of heat. Analysis of the crude reaction product shows no side products, and no decomposition of the *N*-chloromorpholine product.

When the same reaction was carried out using a toluene–water biphasic system, the calorimeter trace was more complex with mass transfer effects making interpretation difficult (Supporting Information File 1).

The calorimetric analysis shows chloramine formation to be an energetic process with a significant associated exotherm illustrating the need for efficient temperature control during the reaction to ensure a safe process. In batch it would be necessary to cool the reaction with an ice bath or similar, however, the continuous reactors employed in this work, with increased surface area-to-volume ratio, allows effective heat dissipation under ambient conditions.

### Continuous reactor

**Figure 2:**
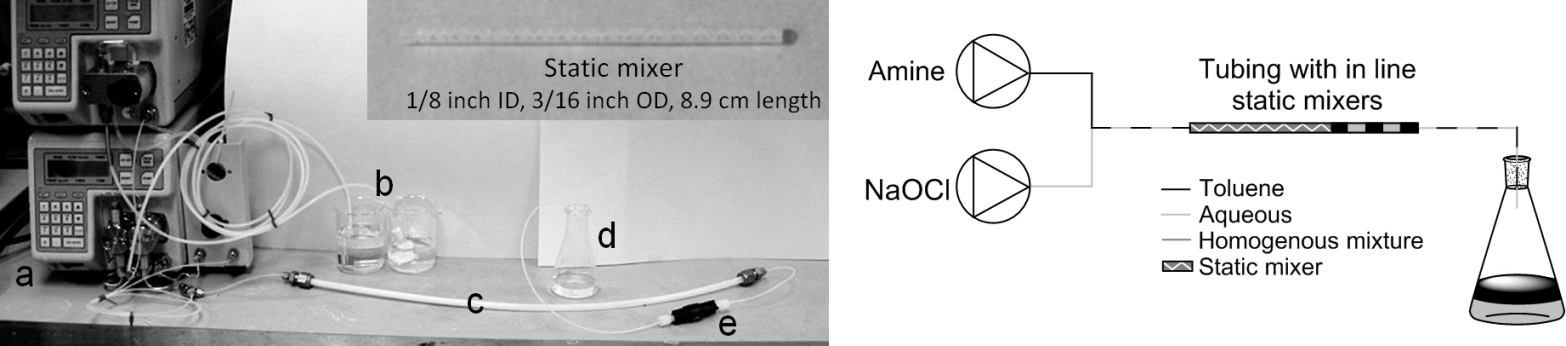
Meso-scale static mixer set-up for continuous *N*-chloramine formation. (a) Pumps, (b) reagent solution reservoirs, (c) reactor tube containing static mixers, (d) collection vessel, (e) back pressure regulator (75 psi).

A nylon/PTFE tubular reactor was constructed that incorporates static mixers for enhanced mixing of the biphasic reaction solution, made of the acetal homopolymer, Delrin^®^ that are solvent and oxidant resistant [34]. The set-up comprised one pump for the organic phase (amine/toluene) and one for the aqueous phase (NaOCl/water). The feeds were connected via a stainless steel T-piece to tubing (1/4 inch OD, 3/16 inch ID) containing static mixer tubes (1/8 inch ID, 3/16 inch OD) along the flow channel (Figure 2). The length of the reactor and number of static mixer inserts were adjusted to vary the residence time, thus maintaining sufficient flow rate to give effective mixing.

**Figure 3:**
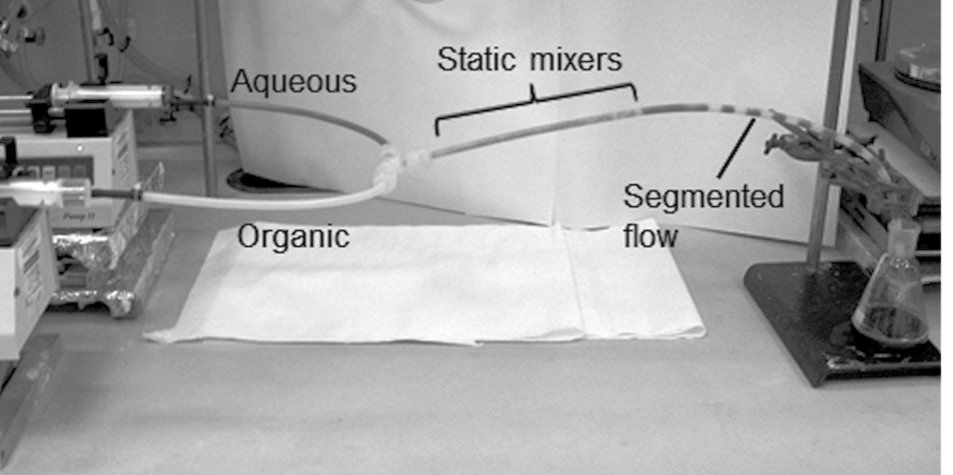
Effect of static mixers on biphasic solution.

Initial experiments using toluene and an aqueous dye were used to assess static mixer performance. Figure 3 shows the solutions being pumped simultaneously into a T-piece with equal flow rates. As the solution progresses into the tube containing the static mixers, it can be seen to be a well-mixed emulsion. Shortly after emanating from the mixed region, the biphase separates into a segmented flow before being collected into a flask.

**Figure 4:**
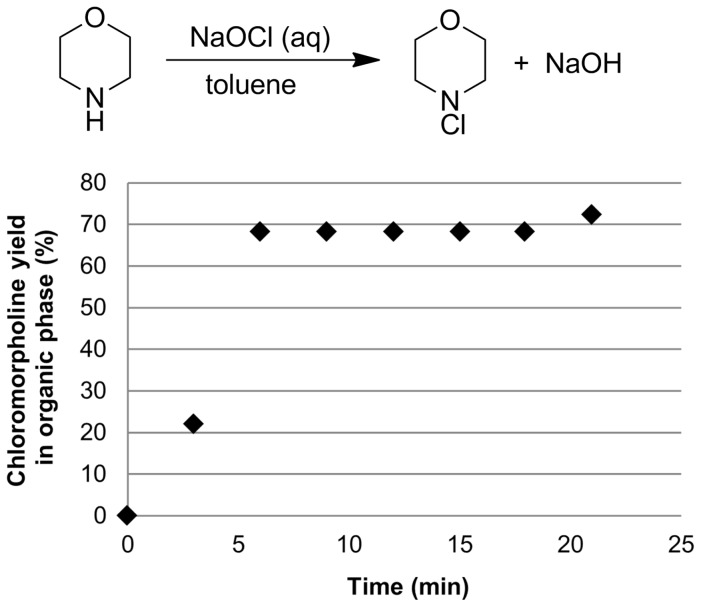
Progress of reaction for continuous formation of *N*-chloromorpholine. Morpholine (toluene) 0.9 M 1 mL/min, NaOCl (aq) 0.9 M, 1.1 mL/min, 6 mL total reactor volume, of which 0.8 mL is static mixers. Residence time = 2.9 min. Conversion refers to composition of organic phase of reaction solution.

Initial reactions looked at the effect of increasing the number of static mixers within the reactor and also increasing residence time by decreasing the flow rate of reagents. The progress of the reaction was studied to determine the point at which steady state is achieved and to assess the consistency of the reaction over several reactor volumes (Figure 4). The reaction reaches steady state after 6 minutes (2 residence times) with fairly consistent conversion achieved thereafter. The *N*-chloromorpholine yield was measured by direct sampling of the toluene solution and measuring by quantitative ^1^H NMR.

Table 1 entry 1 shows the poor conversion observed using the T-piece alone, whilst higher conversions are seen with increased static mixed volume. Table 1 entry 2 shows higher chloramine formation with increased residence time (*T*_res_) as a result of lower flow rates, with a maximum 89% conversion at a *T*_res_ of 20 min. Even at flow rates of 0.15 mL/min there appears to be intimate mixing of the aqueous–organic phases. Both intense mixing and long residence times are required because the *N*-benzylmethanamine partitions mainly in the organic phase with *K*_D_ [organic]/[aqueous] = 28.8, causing the reaction to be limited by the mass transfer rate. For example mixing in the T-piece alone gives 11% conversion with *N*-benzylmethanamine, but 46% with 1,4-morpholine; also 65% conversion is seen with 0.8 min of intense mixing of *N*-benzylmethanamine with NaOCl, whilst 68% is seen with 1,4-morpholine mixed for half this time, because it partitions more favourably into the aqueous phase with *K*_D_ = 0.01.

**Table 1:** Effect of reactor parameters on chloramine formation.^a^

Entry	Product	NaOCl (equiv)	Mixed volume (mL)	Total RV (mL)	*T*_res_ (min)	Amine conv. (%)^b^

1	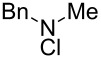	1.1	0^c^ 0^c^ 0.8 1.6	<0.1 6 6 6	<0.05 3 3 3	11 39 57 65

2	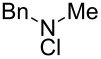	1.1	1.6	6	1.2^d^ 3 6^e^ 12^f^ 20^g^	41 65 69 78 89

3	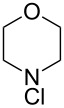	1.1	0^c^ 0.8	<0.1 6	<0.05 3	46 68

^a^*T*_res_ = residence time, RV = reactor volume. Reaction conditions: 1 M amine in toluene and 1.1 M NaOCl (aq) at equal flow rates of 1 mL/min, room temperature. ^b^Steady state conversion by NMR vs internal standard. ^c^T-piece only. ^d^Flow rates = 2.5 mL/min. ^e^Flow rates = 0.5 mL/min. ^f^Flow rates = 0.25 mL/min. ^g^Flow rates = 0.15 mL/min.

### Substrates

**Figure 5:**
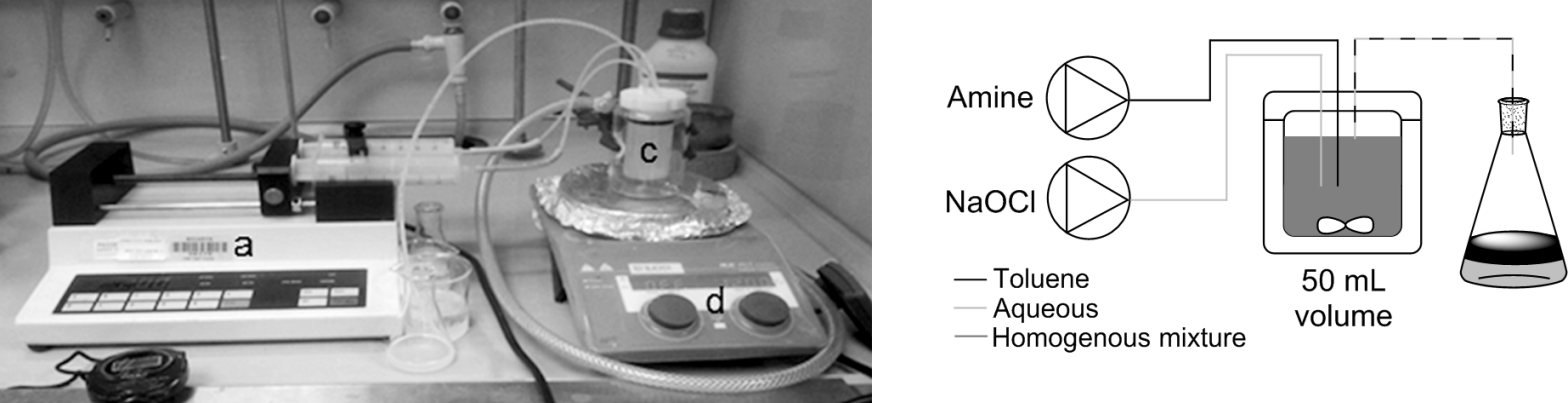
CSTR set-up for *N*-chloramine formation. (a) Syringe pump, (b) collection vessels, (c) reactor (50 mL), (d) stirrer plate: stirring rate 1200 rpm.

**Figure 6:**
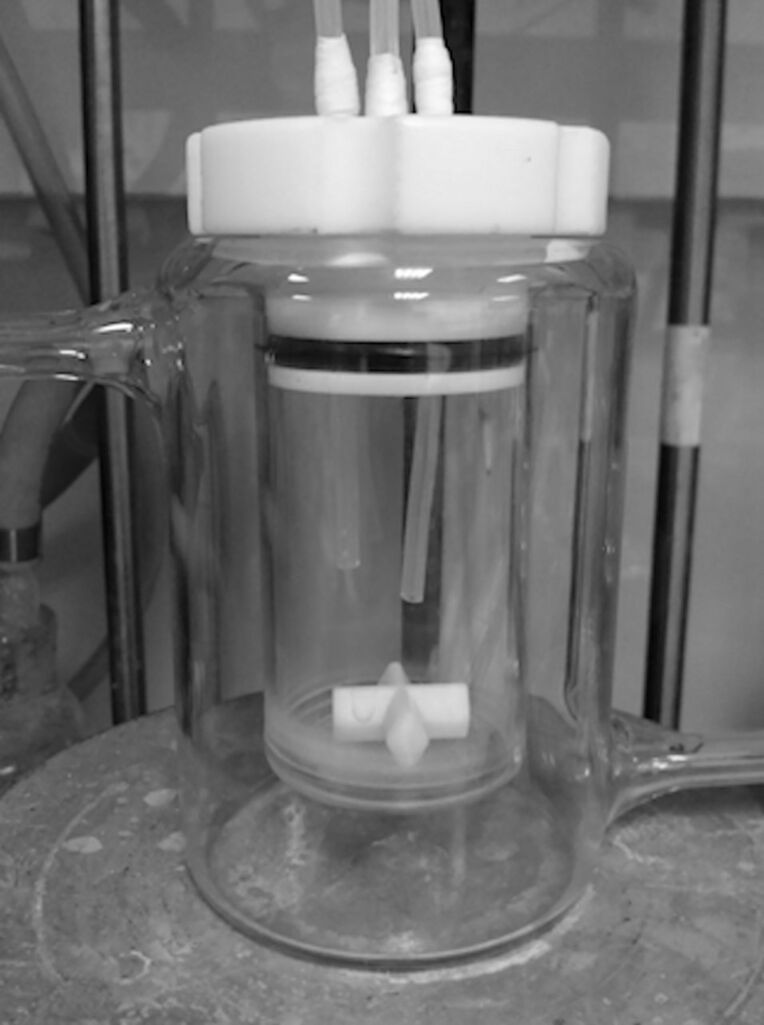
Interior of 50 mL CSTR.

The formation of a range of *N*-chloro-*N*,*N*-dialkylamines was investigated. Some were found to react relatively slowly, partly for the mass transfer reasons discussed above, and possibly for electronic and steric reasons as well. The tube reactor did not conveniently allow sufficient residence times for full conversion of amine to chloramine to be achieved, and in such cases a continuous stirred tank reactor (CSTR), able to provide longer residence times, was used instead (Figure 5 and Figure 6).

Table 2 shows reaction parameters investigated for the reaction of different amines: residence time, reactor volume, number of static mixers and molar equivalents of NaOCl. Formation of *N*-chloromorpholine and *N*-chloropiperidine (Table 2, entries 1 and 2) proceeded with high yields under short reaction times using the static mixers. *N*-chloro-*N*-methyl-*p*-toluenesulfonamide and *N*-chloro-*N*-benzylmethanamine proceeded with good yields in both the tube reactor and CSTR, however, for the tube reactor 1.5 equiv NaOCl were required for complete reaction of *N*-methyl-*p*-toluenesulfonamide, and 20 min residence time was required for high yields of *N*-chloro-*N*-benzylmethylamine. Due to the low solubility of *N*-methyl-*p*-toluenesulfonamide in toluene the tube reactor was susceptible to blockage. This was overcome using EtOAc as the organic phase, and gave more consistent results. Formation of *N*-chlorodibutylamine proceeded slowly within the tube reactor and the longer residence times leant themselves to the CSTR. Table 2 entry 8 shows the need for 2 equiv NaOCl with 20 min residence time to give 35% conversion of the chloramine product with the tubular reactor. However, the conversion was improved markedly using the CSTR and enabled use of only 1.1 equiv of NaOCl to give quantitative formation of the product. The reaction of dibenzylamine with NaOCl proceeds slowly within the CSTR achieving only 40% conversion in 50 min, which may reflect its lower reactivity, since the partition coefficient is similar to that of dibutylamine. The productivities of *N*-chloramine formation are in the range of 0.04–0.06 mol/h.

**Table 2:** Formation of secondary chloramines.^a^

Entry	Product	*K*_D_	Reactor type	Reactor vol. (mL)^b^	*T*_res_ (min)	Amine conv. (%)^c^	Yield (%)^c^

1^d^	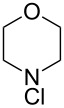	0.01	Static mixers	6 (0.8)	4	100	84

2^e^	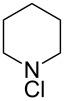	0.84	Static mixers	4 (1.6)	4	100	94

3^f^	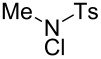	28.9	Static mixers	6 (1.6)	3	97	100
4	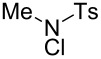	28.9	CSTR	50	50	100	72–98^g^

5	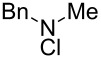	28.8	Static mixers	6 (1.6)	20	89	87
6	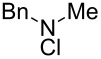	28.8	CSTR	50	25	100	100

7^h^	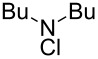	118	Static mixers	4 (1.6)	20	35	Not determined
8	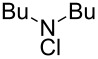	118	CSTR	50	50	100	100

9	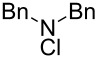	114	CSTR	50	50	40^h^	Not determined

^a^*K*_D_ = [amine] in organic/[amine] in aqueous phase. Reaction conditions: Equal flow rates of amine and NaOCl solutions used. 1 M amine in toluene and 1.1 M NaOCl (aq) were used. Reactions conducted at room temperature. ^b^Static mixed volume in parentheses. ^c^Determined by NMR vs internal standard. ^d^NaOCl (aq) 2.2 M, flow rate 0.5 mL/min, amine 1 M in toluene, flow rate 1 mL/min. ^e^1.5 M NaOCl (aq). ^f^EtOAc solvent instead of toluene used due to solubility of amine. ^g^Low solubility of starting material in toluene caused problems with amine feed giving variation in yields. ^g^2 M NaOCl (aq) used. ^h^40% *N*-chlorodibenzylamine and 60% residual dibenzylamine observed by ^1^H NMR, no other products observed.

### Green metrics

A key driver within the chemical industry is the need for greener, more sustainable processes. In order to assess the sustainability of the continuous process for chloramine formation described in this publication, the metrics of the reaction were assessed and compared with existing literature procedures [35]. The results are summarised in Table 3.

**Table 3:** Comparison of green metrics for different chloramine formation procedures.

Entry	1^a^	2^b^	3^c^	4^d^	5^e^

Amine	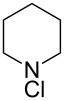	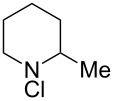	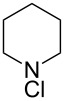	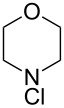	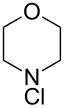
Chlorine source	NaOCl	NaOCl/ *t-*BuOH	*N*-chloro succinimide	NaOCl	NaOCl
Reactor	Flow	Batch	Batch	Batch	Flow
Reaction solvent	toluene, H_2_O	TBME, H_2_O	Et_2_O^f^	H_2_O	toluene, H_2_O
Work-up solvents	–	H_2_O, brine	H_2_O	Et_2_O	–
Conversion	100	100	100	100	100
Yield	94	100	90	88	84
Reaction mass efficiency	57.73	63.42	46.37	63.44	60.24
Atom economy	74.94	53.94	54.69	75.25	75.25
Mass intensity: total	18.17	37.5	80.3	12.39	15.09
Mass intensity: reaction	18.17	7.56	33.71	8.43	15.09
Mass intensity: reaction chemicals	1.73	2.03	2.16	1.58	1.66
Mass intensity: reaction solvents	16.43	5.54	31.55	6.85	13.43
Mass intensity: work-up	–	29.94	46.58	3.96	–

^a^See Table 2, entry 3. ^b^Reference [20]. ^c^Reference [36]. ^d^Reference [11]. ^e^See Table 2, entry 1. ^f^DCM has also been used for reactions of other amines with NCS [3].

The yields of the continuous *N*-chloramine process were high, and comparable to literature batch procedures. The atom economy of our process was increased compared to the use of *N*-chlorosuccinimide [3], or NaOCl/*t-*BuOH, Table 3, entries 2 and 3 [20]. There are also improvements to the total mass intensity of the flow procedure, primarily by avoiding work-up and purification procedures. The mass efficiency remains low, but is comparable to literature procedures. Whilst increasing the reactant concentrations is possible, the safe removal of the heat of reaction must be considered, moreover the flow process is much more productive than batch. Nevertheless the *N*-chloramine solution that is generated should be used directly, so is ideally coupled with a second flow process, and the results of this will be reported elsewhere.

## Conclusion

The facile synthesis of *N*,*N*-dialkyl-*N*-chloramines is described using either a tubular reactor with static mixers or a continuous stirred tank reactor; both are able to promote efficient mixing of the biphasic reaction solution. Those amines which partition mainly in the organic phase required longer reaction times to compensate for the reduced mass transfer between the organic and aqueous phases, and good yields with useful productivities were achieved for most. The green metrics of the reaction have been assessed and compared to existing procedures for chloramine formation, and have shown the continuous process is improved over previous procedures. Work to expand the scope of *N*-chloramines, and their subsequent use in flow reactions with alkenes to make amines, aldehydes to make amides, and base to make imines is ongoing.

## Experimental

All reagents were used as received from suppliers without purification. Sodium hypochlorite solution 10–15% available chlorine was purchased from Sigma-Aldrich. The accurate NaOCl concentration was determined by titration (Supporting Information File 1) and diluted to the required concentration with distilled water. CDCl_3_ purchased from Sigma-Aldrich was used for NMR analysis. NMR spectra (^1^H and ^13^C) were obtained on either a Bruker Advance 500 MHz, 400 MHz or a Bruker DPX 300 MHz spectrometer. NMR spectra were referenced to either TMS or CHCl_3_. The partitioning of the amine reagents in the organic phase of a biphasic organic/aqueous solution was determined by GC analysis using an Agilent HP 6890 with FID (Supporting Information File 1). Calorimetry experiments were carried out using HEL AutoMATE parallel reactors with HEL WinISO 2225 and HEL IQ 1.2.16 software. For the tube reactor, Harvard syringe pumps (model 981074) or JASCO PU-980 HPLC pumps were connected via a syringe and 1.5 inch, 21 guage disposable needle to PTFE tubing with 1/16 inch OD. The 1/16 inch stainless steel T-piece and stainless steel 1/16 inch to 1/4 inch increasing connector were obtained from Swagelok. The reactor tube comprises of PTFE tubing 1/4 inch OD and in-line plastic static mixers supplied by Nordson EFD (3/16 inch OD, mixer element diameter 1/8 inch, 3.5 inch length). For the CSTR, the same Harvard syringe pumps were used along with PTFE tubing with 1/8 inch OD.

### General procedure for *N*-chloramine formation using the tube reactor and static mixers

The required number of static mixers (3/16 inch OD, 1/8 inch mixing element diameter 3.5 inch length) was inserted into a length of 1/4 inch PTFE tubing to give the overall required reactor volume. The reactor was connected to 1/16 inch tubing via a 1/4–1/16 inch reducing adapter. The tubing was split with a T-piece to 2 pumps (either piston or syringe pumps). A 1 M solution of amine in toluene was prepared and fed via pump 1 at the required flow rate. A 1.1 M solution of aqueous NaOCl was fed via pump 2 at the required flow rate. The reaction solution was flowed through the reactor and collected in residence time fractions. The organic phase was separated from each fraction to avoid further interaction with the NaOCl solution. The organic phase was analysed by ^1^H NMR. For non-volatile products yields were obtained by removal of toluene from the organic phase of each residence time fraction and weighing the resulting product. For volatile products yields were obtained by ^1^H NMR analysis. 100 μL or the organic phase was weighed and analysed by ^1^H NMR. The ratio of toluene/starting material/product and mass of 100 μL of the solution was used to determine the *N*-chloramine concentration in the solution. From this the yield of *N*-chloramine in the full organic phase could be determined.

### General procedure for *N*-chloramine formation using the CSTR

Through the lid of a 50 mL jacketed glass vessel containing a stirrer bar was inserted 2 lengths of 1/8 inch PTFE tubing connected to syringe pumps. A third length of tubing was inserted so that it ended flush with the base of the lid to act as an overflow tube out of the reactor into a collection vessel. The reactor was secured on a stirrer plate.

A 1 M solution of amine in toluene was prepared and fed via pump 1 at the required flow rate. A 1.1 M solution of aqueous NaOCl was fed via pump 2 at the required flow rate. The reaction solution was flowed into the reactor, and once full was eluted from the reactor via the overflow tube and collected. The solution was collected in residence time fractions. The organic phase was separated from each fraction to avoid further interaction with the NaOCl solution. The organic phase was analysed by ^1^H NMR. For non-volatile products yields were obtained by removal of toluene from the organic phase of each residence time fraction and weighing the resulting product. For volatile products, yields were obtained by ^1^H NMR analysis. 100 μL or the organic phase was weighed and analysed by ^1^H NMR. The ratio of toluene/starting material/product and mass of 100 μL of the solution was used to determine the *N*-chloramine concentration in the organic solution. From this the yield of *N*-chloramine in the full organic phase could be determined.

### *N*-Chloro-1,4-morpholine

Prepared according to the general procedure using the tube reactor with 2 static mixers, a 6 mL reactor volume and a residence time of 4 min, 2.2 M NaOCl (aq) at 0.5 mL/min and 1 M morpholine in toluene at 1 mL/min. Due to the high volatility of *N*-chloromorpholine and its reported instability the product was not isolated and was instead obtained as a solution in toluene (0.84 M, 84%) by separation of the organic phase form the aqueous phase of the reaction solution. The yield of product was determined by NMR analysis as in the typical procedure for volatile products. The toluene solution was analysed by NMR and data matches that reported in the literature [17]. ^1^H NMR (CDCl_3_, 500 MHz) δ ppm 3.69 (br s, 4H, C*H*_2_OC*H*_2_), 3.12 (br s, 4H C*H*_2_NClC*H*_2_); ^13^C NMR (CDCl_3_, 125 MHz) δ ppm 67.72 (*C*H_2_O*C*H_2_), 63.03 (*C*H_2_NCl*C*H_2_).

### *N*-Chloropiperidine

Prepared according to the general procedure using the tube reactor with 4 static mixers, a 4 mL reactor volume and a residence time of 4 min, 1.5 M NaOCl (aq) at 0.5 mL/min and 1 M piperidine in toluene at 0.5 mL/min. Due to the high volatility of *N*-chloropiperidine the product was not isolated and was instead obtained as a solution in toluene (0.94 M, 94%) by separation of the organic phase from the aqueous phase of the reaction solution. The yield of product was determined by NMR analysis as in the typical procedure for volatile products. The toluene solution was analysed by NMR and data matches that reported in the literature [17]. ^1^H NMR (CDCl_3_, 300 MHz) δ ppm 3.49–2.63 (br m, 4H, C*H*_2_NClC*H*_2_), 1.84 (quin, *J* = 5.8 Hz, 4H, 2 x CH_2_C*H*_2_CH_2_), 1.75–1.32 (br m, 2H, NCH_2_CH_2_C*H*_2_); ^13^C NMR (CDCl_3_, 125 ppm) δ ppm 64.00 (2 × *C*H_2_NCl), 27.68 (2 × *C*H_2_CH_2_NCl), 23.07 (*C*H_2_(CH_2_)_2_NCl).

### *N*-Benzyl-*N*-chloromethanamine

Prepared according to the general procedures. 1) Tube reactor with 4 static mixers, a 6 mL reactor volume and a residence time of 3 min, 1.1 M NaOCl (aq) at 0.15 mL/min and 1 M *N*-benzylmethylamine in toluene at 0.15 mL/min. 2) CSTR with a reactor volume of 50 mL, and residence time of 25 min. 1.1 M NaOCl (aq) at 1 mL/min and 1 M *N*-benzylmethylamine in toluene at 1 mL/min were used. Due to the volatility of *N*-benzyl-*N*-chloromethanamine the product was not isolated and was instead obtained as a solution in toluene (1 M, quantitative yield) by separation of the organic phase from the aqueous phase of the reaction solution. The yield of product was determined by NMR analysis as in the typical procedure or volatile products. The toluene solution was analysed by NMR and data matches that reported in the literature [17]. ^1^H NMR (CDCl_3_, 300 MHz) δ ppm 7.32–7.28 (m, 5H, C*H*Ar), 3.99 (s, 2H, C*H*_2_), 2.88 (s, 3H, C*H*_3_); ^13^C NMR (CDCl_3_, 125 MHz) δ ppm 139.37 (*C*Ar), 128.58 (2 × *C*HAr), 128.49 (2 × *C*HAr), 127.32 (*C*HAr), 55.85 (*C*H_2_), 35.68 (*C*H_3_).

### *N*-Chloro-*N*-methyl-*p*-toluenesulfonamide

Prepared according to the general procedures. 1) Tube reactor with 4 static mixers, a 6 mL reactor volume and a residence time of 3 min, 1.5 M NaOCl (aq) at 1 mL/min and 1 M *N*-benzylmethylamine in EtOAc at 1 mL/min. 2) CSTR with a reactor volume of 50 mL, and residence time of 25 min. 1.1 M NaOCl (aq) at 1 mL/min and 1 M *N*-benzylmethylamine in toluene at 1 mL/min were used. The product was isolated for each reactor volume by separation of the organic phase for each reactor volume of solution and removal of the solvent by rotary evaporation to give a white solid (1 reactor volume gives 666 mg, 3.0 mmol, quantitative yield). NMR data matches that reported in the literature [37]. ^1^H NMR (CDCl_3_, 500 MHz) δ ppm 7.82 (d, *J* = 8.4 Hz, 2H, C*H*Ar), 7.42 (d, *J* = 8.4 Hz, 2H, C*H*Ar), 3.09 (s, 3H, NC*H*_3_), 2.48 (s, 3H, Ar-C*H*_3_); ^13^C NMR (CDCl_3_, 125 MHz) δ ppm 145.75 (*C*Ar), 129.80 (2*C*HAr), 129.78 (2 *C*HAr), 128.28 (*C*Ar), 45.51 (N*C*H_3_), 21.70 (Ar*C*H_3_).

### *N*-Chloro-*N*,*N*-dibutylamine

Prepared according to the general procedures. 1) Tube reactor with 4 static mixers, a 4 mL reactor volume and a residence time of 20 min, 2 M NaOCl (aq) at 0.1 mL/min and 1 M dibutylamine in toluene at 0.1 mL/min. 2) CSTR with a reactor volume of 50 mL, and residence time of 50 min. 1.1 M NaOCl (aq) at 0.5 mL/min and 1 M dibutylamine in toluene 0.5 mL/min were used. The product was isolated for each reactor volume by separation of the organic phase for each reactor volume of solution and removal of the solvent by rotary evaporation to give a colourless oil (1 reactor volume gives 8.17 g, 50 mmol, quantitative yield). NMR data matches that reported in the literature [17]. ^1^H NMR (CDCl_3_, 300 MHz) δ ppm 3.07–3.02 (m, 4H, 2 × NClC*H*_2_), 1.85–1.74 (m, 4H, 2 × NCH_2_C*H*_2_CH_2_), 1.54–1.45 (m, 4H, 2 × C*H*_2_CH_3_), 1.11–1.05 (t, *J* = 7.4 Hz, 6H, 2 × C*H*_3_); ^13^C NMR (CDCl_3_, 125 MHz) δ ppm 64.04 (2 × *C*H_2_NCl), 30.01 (2 × *C*H_2_CH_2_NCl), 20.03 (2 × *C*H_2_(CH_2_)_2_NCl), 13.90 (2 × *C*H_3_).

### *N*-Chloro-*N*,*N*-dibenzylamine

Prepared according to the general procedure using the CSTR with a reactor volume of 50 mL, and residence time of 50 min, 1.1 M NaOCl (aq) at 0.5 mL/min and 1 M dibenzylamine in toluene 0.5 mL/min were used. The product was not isolated and was instead obtained as a solution in toluene (0.4 M, 40% conversion) by separation of the organic phase from the aqueous phase of the reaction solution and removal of the solvent by rotary evaporation. The conversion to product was determined by NMR analysis of dried product (orange oil) and data obtained matches that reported in the literature [38]. ^1^H NMR (CDCl_3_, 300 MHz) 40% conversion to chloramine δ ppm 7.56–7.44 (m, 20H, C*H*Ar in amine and chloramine), 4.28 (s, 4H, 2 × NClC*H*_2_ in chloramine), 3.80 (s, 4H 2 × NHC*H*_2_ in amine); ^13^C NMR (CDCl_3_, 125 MHz) δ ppm chloramine: 137.17 (2 × *C*Ar), 129.18 (4 × *C*HAr), 128.52 (4 × *C*HAr), 127.95 (2 × *C*HAr), 67.24 (2 × *C*H_2_NCl); amine: 136.26 (2 × *C*Ar), 128.47 (4 × *C*HAr), 128.39 (4 × *C*HAr), 127.19 (2 × *C*HAr), 52.94 (2 × *C*H_2_NH).

## Supporting Information

File 1 Details of the titration method for determination of NaOCl strength, determination of amine partition coefficients, GC analytical conditions and calorimetry.
