# Supplementary material for: Continuous formation of N-chloro-N,N-dialkylamine solutions in well-mixed meso-scale flow reactors
Source: Beilstein J Org Chem. 2015 Dec 2;11:2408–17. doi: 10.3762/bjoc.11.262 (PMC4685760; doi:10.3762/bjoc.11.262)
Supplement: File 1 — Details of the titration method for determination of NaOCl strength, determination of amine partition coefficients, GC analytical conditions and calorimetry. [file Beilstein_J_Org_Chem-11-2408-s001.pdf]

Supporting Information

for

Continuous formation of *N*-chloro-*N*,*N*-dialkylamine solutions in well-mixed meso-scale flow reactors

A. John Blacker*[1] and Katherine E. Jolley[1]

Address: [1] Institute of Process Research and Development, School of Chemistry and School of Chemical and Process Engineering, University of Leeds, Woodhouse Lane, Leeds, LS2 9JT, Leeds, UK

Email: A. John Blacker* - j.blacker@leeds.ac.uk

* Corresponding author

Details of the titration method for determination of NaOCl strength, determination of amine partition coefficients, GC analytical conditions and calorimetry

Titration of NaOCl (aq)

1 mL acetic acid and 0.35 g of KI was diluted to 50 mL with distilled water. To the solution was added 1 mL of the NaOCl solution obtained from Sigma-Aldrich. 0.1 M $Na_2S_2O_3$ (aq) was then titrated into the aqueous solution of KI, acetic acid and NaOCl until the orange solution becomes colourless. The concentration of the initial NaOCl solution can thus be determined using the equations below.

$$^{-}OCl + 2I^{-} \longrightarrow I_2 + Cl^{-}$$

$$I_2 + 2S_2O_3^{2-} \longrightarrow S_4O_6^{2-} + 2I^{-}$$

Determination of fraction of amine partitioning in the organic phase of a biphasic organic/aqueous solution

100 µL of amine (when liquid at room temperature) or 100 mg of amine (when solid at room temperature) was added to a biphasic solution comprising of 1 mL of the organic solvent (toluene or EtOAc) and 1 mL water. The resulting solution was mixed thoroughly and then allowed to stand and the phases separate. 100 µL of the organic and aqueous phases of each solution were in turn added to 1 mL of MeOH and analysed by GC. The concentration of amine in each phase was determined and used to calculate K_D using the equation below:

$$K_D = \frac{[amine]\ in\ organic}{[amine]\ in\ aqueous}$$

GC Analysis of amines

GC Method: HP-5 column 30 m × 0.32 mm × 0.25 µm, H_2 10 psi, split ratio 5:1, T = 35 °C 3 min, 10 °C/min to 200 °C, 200 °C 3 min, 280 °C 3 min.

Morpholine: 2.2 min

Piperidine: 1.8 min

N-Benzylmethylamine: 7.1 min

N-Methyl-*p*-toluenesulfonamide: 6.1 min

Dibutylamine: 3.4 min

Dibenzylamine: 16.2 min

Toluene: 1.5 min

Calorimetry

1. To a 50 mL vessel was added 20 mL of 1 M solution of morpholine in water. 1.1 M NaOCl (aq) was then fed into the morpholine solution according to the required plan as detailed below.

To determine the enthalpy of the reaction, power compensation calorimetry was used with the reaction plan as detailed below.

Steady State 1:
Stir 300 rpm
Reactor temperature: 5 °C
Circulator temperature: −15 °C
Time: 60 min

Feed Step:
Stir 300 rpm
Reactor temperature: 5 °C
Circulator temperature: −15 °C
Feed 1.1 M NaOCl (aq) at 1 mL/min for 20 min (20 mL, 22 mmol NaOCl)
Time: 20 min

Steady State 2:
As for Steady State 1

The enthalpy (kJ mol^{-1}) of the reaction was determined using IQ analytical software.

2. To a 50 mL vessel was added 20 mL of 1 M solution of morpholine in toluene.

To determine the enthalpy of the reaction, power compensation calorimetry was used with the reaction plan as detailed below:

Steady State 1:
Stir 300 rpm
Reactor temperature: 5 °C
Circulator temperature: −15 °C
Time: 60 min

Feed Step:
Stir 300 rpm
Reactor temperature: 5 °C
Circulator temperature: −15 °C
Feed 1.1 M NaOCl (aq) at 1 mL/min for 20 min (20 mL, 22 mmol NaOCl)
Time: 20 min

Steady State 2:
As for Steady State 1

The enthalpy (kJ mol^{-1}) of the reaction was determined using IQ analytical software.

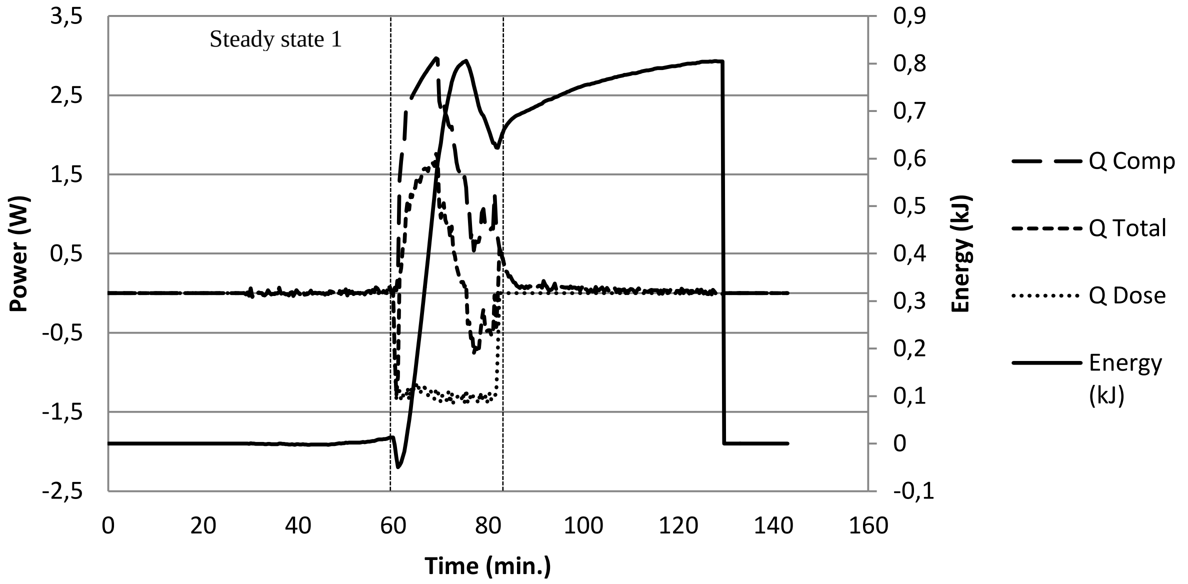

Figure 1: Calorimeter trace for biphasic reaction of morpholine (toluene) and NaOCl (aq). Q Comp: compensatory power, Q Total: total power, Q Dose: power delivered

by dosing of room temperature NaOCl (aq) to cooled reaction solution, Energy: heat energy.

3. To determine the adiabatic temperature rise, 1.1 M NaOCl (aq) was fed into 1 M morpholine in toluene. The following reaction plan was used.

Steady State 1:
Stir 300 rpm
Reactor temperature: room temperature, no temperature control used
Time: 60 min

Feed Step:
Stir 300 rpm
Reactor temperature: room temperature, no temperature control used
Feed 1.1 M NaOCl (aq) at 1 mL/min for 20 min (20 mL, 22 mmol NaOCl)
Time: 20 min

Steady State 2:
As for Steady State 1

The temperature rise due to the reaction was determined (Figure 2) by comparison of the initial temperature at the start of the feed step and the maximum temperatures reached during the feed (Experiment 1). The control reactions for the equivalent additions of water to toluene; water to morpholine/toluene and NaOCl aq. to toluene, the ΔH was found to be negligible in all cases by calorimetry (eg. Experiment 2).

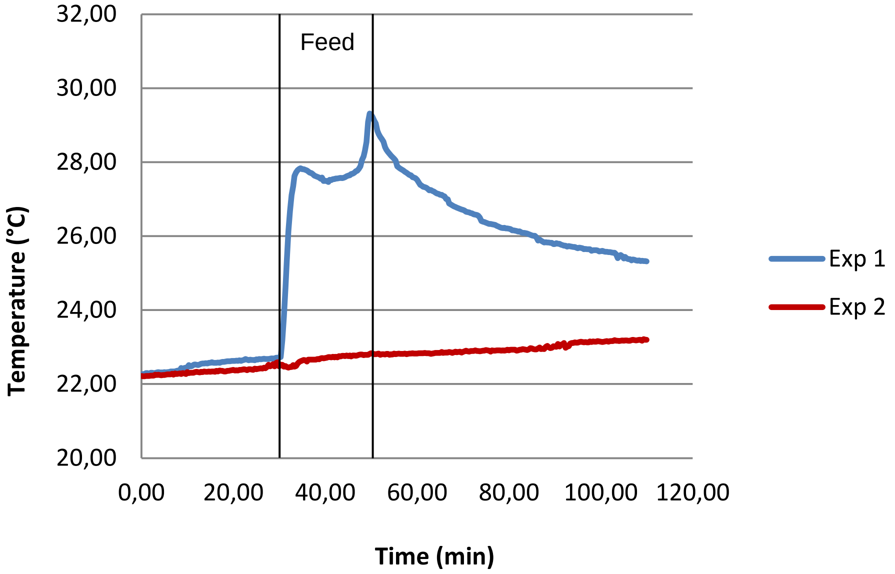

Figure 2: Temperature rise for addition of room temperature NaOCl (aq) to room temperature morpholine (toluene) (Exp 1) and addition of room temperature water to room temperature toluene (Exp 2).

Calculation of enthalpy change of reaction

Proposed mechanism for chrloamine formation [1].

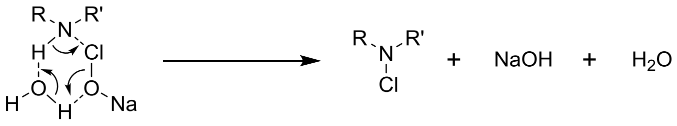

Average bond enthanlpies [2]:

Bond formation	Bond breakage
N–Cl 200 $kJmol^{-1}$	N–H 388 $kJmol^{-1}$
O–H 463 $kJmol^{-1}$	O–H 463 $kJmol^{-1}$
O–H 463 $kJmol^{-1}$	O–Cl 203 $kJmol^{-1}$

$$\Delta H_{rxn} = (388 + 463 + 203) - (200 + 463 + 463)\ kJ\ mol^{-1}$$
$$= -72\ kJ\ mol^{-1}$$

References

1. Weil, I.; Morris, J. C. *Journal of the American Chemical Society* **1949,** *71,* 1664-1671.

2. Atkins, P.;Overton, T.;Rourke, J.;Weller, M.;WArmstrong, F. *Inorganic Chemistry*, Eds 4; Vol. Oxford University Press: Oxford, UK, 2006.
